# Dairy Sites with Milk Exposure Are Most Likely to Have Detection of Influenza A Virus

**DOI:** 10.3390/microorganisms14030584

**Published:** 2026-03-05

**Authors:** Chloe Stenkamp-Strahm, Brian McCluskey, Blaine Melody, Natalie Urie, Nicole Amey, Richanne Lomkin, A. J. Campbell, Seema Lakdawala, Jason Lombard

**Affiliations:** 1 Department of Clinical Sciences, College of Veterinary Medicine and Biomedical Sciences, Colorado State University, Fort Collins, CO 80523, USAjason.lombard@colostate.edu (J.L.); 2 Department of Veterinary Clinical and Life Sciences, College of Veterinary Medicine, Utah State University, Logan, UT 84322, USA; 3 Lonestar Epidemiology Consulting, Livermore, CO 80536, USA; 4 Lander Veterinary Clinic, 4512 S Walnut Rd., Turlock, CA 95380, USA; 5 United States Department of Agriculture, Animal and Plant Health Inspection Service, East Lansing, MI 48823, USA; 6 United States Department of Agriculture, Animal and Plant Health Inspection Service, Lakewood, CO 80401, USA; 7 Department of Microbiology and Immunology, Emory University, Atlanta, GA 30322, USA

**Keywords:** influenza A virus, dairy, environment, surveillance

## Abstract

Highly pathogenic avian influenza virus of the H5N1 subtype has been infecting U.S. dairy cattle and spreading among dairy operations since March 2024. H5N1 surveillance systems for dairies are needed, but information on whether environmental sampling can inform these systems is lacking. To guide a surveillance framework, we determined the environmental sites with Influenza A virus (IAV) detection on H5N1-affected dairies (n = 25) in four states (California, Colorado, Michigan, and Ohio) and explored sample characteristics that may have influenced detection. A total of 581 samples from dairy environmental sites were characterized for IAV RNA via rRT-PCR, and classified into six categories. A total of 94 samples (16.2%) had IAV detected, and the Ct values measured from these samples were typically higher than those measured in bulk tank milk from a subset of sampled herds. A majority of IAV detections were made from the following site categories: milking equipment/personal protective equipment, parlor surfaces, and wastewater/lagoons/manure. These results suggest that environmental sites most likely to be contaminated with IAV on dairies are those with exposure to milk. Meanwhile, mixed effect logistic modeling showed that days into an outbreak that samples were collected was associated with IAV detection. These results provide a framework within which to continue the assessment of environmental sampling as a surveillance tool for dairy H5N1. This framework can be strengthened by studies that perform further IAV viral characterization and collect samples from sites prior to, during, and after H5N1 outbreak periods.

## 1. Introduction

In February 2024, a disease syndrome characterized by fever, lethargy, dehydration and an abrupt drop in milk production affected cattle in dairy herds in Texas and Kansas. It was eventually confirmed that this syndrome resulted from an Influenza A virus (IAV) of the H5N1 subtype [1,2,3]. The initial bovine infection likely resulted due to spillover from a wild bird having shed H5N1, with subsequent cow-to-cow transmission occurring among herd mates. Since its discovery in cows, H5N1 clade 2.3.4.4b of both B3.13 and D1.1 genotypes have continued to spread in dairy cattle, with cows on dairy operations in 18 states affected [4,5,6].

During this outbreak, viral RNA has been regularly found in the milk but also variably found in the blood, nasal secretions and urine of clinically ill and nonclinical cows on H5N1-affected dairies [2,7]. Biosecurity recommendations include diverting the milk from sick cows to waste streams, and treating this milk prior to discard [8]. During herd infection, milk and other cattle excretions may serve to contaminate dairy environments with H5N1, and spread the virus to naïve cows within a herd or to other farms via fomites. To date, testing for IAV RNA via real-time reverse transcription polymerase chain reaction (rRT-PCR) in aggregate milk samples from dairy herds (i.e., bulk tank milk) has been leveraged for the surveillance and monitoring of H5N1-affected dairies [9]. These surveillance efforts led to the discovery that the D1.1 genotype, in addition to the B3.13 genotype, had been introduced and was circulating in dairy cattle [6]. Recently, wastewater surveillance in the state of Oregon showed that environmental detections of virus were not likely of dairy origin [10]. Other methods to surveil dairies for H5N1, to the authors’ knowledge, have yet to be investigated.

IAV persistence in the environment outside of animal hosts has been previously studied. IAV has been shown to persist on several non-porous surfaces (steel, tile, rubber and plastic) for up to three days, and on latex for up to six days [11]. A separate study indicated that under the right environmental conditions, H5N1 viruses can persist on certain substrates, such as galvanized metal, glass, and topsoil, for longer than 13 days [12]. Environmental sampling conducted during previous HPAI outbreaks on poultry farms have shown viral detection from air samples [13] and different types of surfaces, including walls/doors, fans, cages, feeding equipment and gloves/masks [14]. Cattle-derived H5N1 strains have been detected and successfully isolated from both rubber and plastic on a dairy in Kansas [15] and were seen to persist experimentally on stainless steel and rubber, the two substrates that comprise commercial milking equipment [16].

Environmental sampling for IAVs has been used within the poultry industries for early detection of HPAI outbreaks, identification of novel viral strains, and measuring success of outbreak intervention strategies [17]. Environmental sampling has been explored for surveillance and the detection of IAVs in swine [18], with environmental sampling showing good detection sensitivity [19]. The utility of using environmental samples for surveillance within the dairy sector, either in lieu of or in addition to bulk tank milk sampling, is unknown, but environmental sampling methods may be convenient and cost-effective.

Our understanding of how the H5N1 virus is transmitted within a dairy herd remains limited [7,20]. Establishing which dairy environmental sites harbor virus during herd outbreaks is advantageous, as it may guide future surveillance efforts and also inform studies that further our understanding of H5N1 transmission. Accomplishing this requires environmental samples taken from affected dairy operations in multiple geographic areas, at different points in their outbreak periods.

The aim of this investigation was to describe detection of IAV RNA in samples taken from multiple sites on dairies affected by H5N1 in California (CA), Colorado (CO), Michigan (MI) and Ohio (OH), and use this information to better understand whether environmental sampling should be prioritized for future dairy H5N1 surveillance.

## 2. Materials and Methods

### 2.1. Design of Investigation

**Figure 1:**
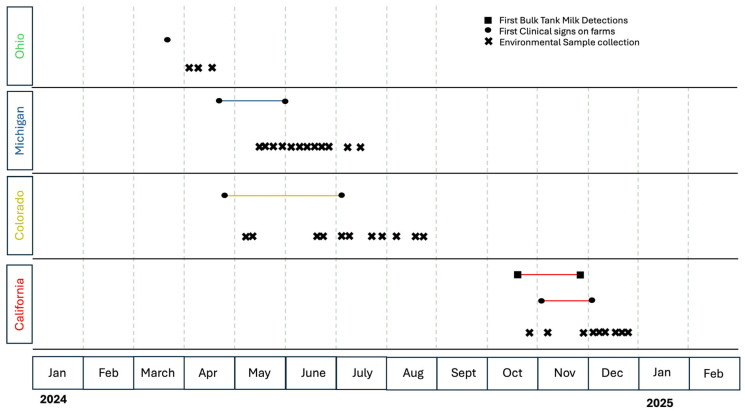
Timeline showing when enrolled dairies had initial clinical signs of H5N1 disease in cows, when bulk tank detections were first made (CA), and when samples were collected from dairy operations in each state.

Because dairy producers have concerns about the regulatory and economic ramification of H5N1 infection, many have been reluctant to participate in outbreak investigations. The samples analyzed in this work were taken from operations that voluntarily agreed to participate in environmental sample collection, and collected as part of outbreak investigations in four states. Figure 1 displays a timeline of when environmental samples were collected, and when dairies in each state experienced initial clinical signs of H5N1 disease in their cattle.

The dates that dairy cattle first experienced clinical signs, used hereafter as the outbreak start dates, were determined via conversations with herd owners/managers, private veterinarians, and completed epidemiologic surveys. Table 1 describes the herd size, cattle breeds and parlor types of participating dairies in each state. More specific information from all enrolled farms was not available (e.g., number of affected cows, management of ill cows, cattle movement data). Specific details regarding sample collection within each state are described below.

In MI, H5N1 in cattle was confirmed on 29 March 2024. A USDA Epidemiological Strike Team was invited by the Michigan Department of Agriculture and Rural Development (MDARD) to study affected dairies and identify potential links for viral dissemination. Twelve dairies in central and southern MI allowed collection of environmental samples from sites on and surrounding their operations. These herds initially experienced clinical signs of the virus between 21 April and 31 May 2024.

In OH, H5N1 in cattle was detected on 25 March 2024. Samples were collected on a single dairy operation starting in early April 2024 by the Ohio Department of Agriculture (ODA) and the United States Department of Agriculture’s Animal and Plant Health Inspection Service, Veterinary Services (USDA:APHIS:VS). This herd experienced clinical signs of H5N1 on 21 March 2024.

In CO, H5N1 was first detected in cattle on 21 April 2024. The Colorado Department of Agriculture (CDA), USDA:APHIS:VS, and Colorado State University (CSU) collected a variety of environmental samples from sites on 6 operations. These herds first experienced clinical signs of H5N1 between 21 April and 7 July 2024. CDA mandated weekly bulk tank milk (BTM) testing of all commercial dairy herds within the state starting in July 2024 [21]. Ct values from this weekly BTM testing were provided to our team, with sample testing beginning 29 July 2024.

In CA, H5N1 was first detected in dairy cattle on 30 August 2024. Lander Veterinary Clinic (LVC) in Turlock, CA, in collaboration with CSU, California Department of Food and Agriculture (CDFA), and USDA:APHIS, collected environmental samples from 6 herds. These herds first experienced clinical signs of H5N1 between 1 November and 1 December 2024. Each CA herd also completed voluntary daily BTM testing for H5N1 starting in the fall of 2024, and prior to herd clinical signs.

All premises enrolled in this investigation were confirmed as infected with IAV H5N1 clade 2.3.4.4b genotype B3.13 via testing of samples from clinically ill cows or BTM at USDA’s National Veterinary Services Laboratories (NVSL) in Ames, Iowa. These confirmations were made prior to environmental sampling for IAV. Data was not collected regarding changes in hygiene or biosecurity that may have been implemented on farms after initial IAV detection.

### 2.2. Environmental and BTM Sample Collection

On each enrolled farm, efforts were made to collect environmental samples from as many sites as possible. Sites sampled included milking parlors (including floors, surfaces, milking equipment, personal protective equipment of workers), housing areas and barns, farm equipment including tractors, implements and other machinery, office surfaces, waterers, feeders, wastewater and lagoon samples. Sterile polyester or cotton-tipped swabs, sterile gauze (3 × 3 inch dimension), or sterile swabs in pre-aliquoted universal viral transport media (UVT; Becton Dickinson (Franklin Lakes, NJ, USA), Cat. 220526) were used to sample surfaces. Each surface was contacted for 10 s during sample collection, although there was some variation in this timing given a need to collect certain samples expediently to not disrupt normal farm operations and milking procedures. Although the exact surface area contacted was not standardized or recorded for each sample, attempts were made to either contact the entire area of interest (e.g., in the case of parlor drains, inflations) or in areas with a large footprint (e.g., housing areas, fences) to not sample beyond a zone of 1 square foot. Dry swabs and gauze were placed in 10–15 mL transport media (brain heart infusion (BHI) or molecular transport media (MTM), provided by NVSL). Lagoon samples were collected by placing a sterile gauze pad or 50 mL conical tube on a weighted fishing rig, casting it into the lagoon, and allowing it to sink. Fluid collected from the saturated gauze or conical tube was then placed into another conical tube. All environmental samples were refrigerated, and then either driven to the lab or shipped on ice overnight.

For the CA dairies, each operation submitted daily BTM samples to LVC. Farms submitted between 1 and 8 of these samples per day, depending on herd size and daily milk production. An aliquot of each sample was tested for IAV RNA via rRT-PCR at Iowa State University Veterinary Diagnostic Laboratory in Ames, IA, USA, after being shipped on ice overnight.

The following National Animal Health Laboratory Network (NAHLN) laboratories completed BTM and/or performed environmental sample processing and testing: Michigan State University Veterinary Diagnostic Laboratory in Lansing, MI, USA, Colorado State University Veterinary Diagnostic Laboratory in Fort Collins, CO, USA, Iowa State University Veterinary Diagnostic Laboratory in Ames, IA, USA, Washington Animal Disease Diagnostic Laboratory in Pullman, WA, USA, Ohio Animal Disease Diagnostic Laboratory in Reynoldsburg, OH, USA, and NVSL (Ames, IA, USA). A subset of environmental samples from California dairies were tested at LVC in Turlock, CA, USA.

### 2.3. Sample Processing, RNA Extraction, rRT-PCR

Samples were preprocessed, RNA was isolated, and rRT-PCR assays for IAV were performed on environmental and BTM samples per NAHLN standard operating procedures and protocols, using a cycle threshold (Ct) cut-off value of 40. Annually, each NAHLN laboratory is required to successfully complete NVSL-administered proficiency tests and quality control procedures for identification of IAV. NAHLN labs use identical protocols, gene targets, reagents and equipment for IAV testing, and protocols and workflows are proprietary within NAHLN agreement with USDA. Virus isolation, whole genome sequencing (WGS), and H5 sample testing were not completed for any of the environmental samples collected in this investigation.

Environmental samples sent to Lander Veterinary Clinic were processed using the following protocol: UVT tubes were vortexed for 10 s with swabs inside and then centrifuged for roughly 10 s to collect liquid at the bottom of each tube. An aliquot was removed for RNA extraction and the remaining sample was stored at 4 °C. RNA extraction was completed using 200–400 μL of sample with a MagMax CORE Nucleic Acid Purification Kit (Applied Biosystems (Foster City, CA, USA), Cat. A32700) paired with the Swine Influenza Virus RNA Test Kit (Applied Biosystems, Cat. 4415200) on a KingFisher Flex 96 system (ThermoFisher, Waltham, MA, USA). A QuantStudio 5 Real-Time PCR System (ThermoFisher, Cat. A28569) was used according to the manufacturer’s instructions to perform rRT-PCR for IAV. Reactions were performed using 8 μL of extracted RNA from each sample.

### 2.4. Analysis

Six sample site categories were created to represent different environmental sampling locations on affected operations. Personal protective equipment (PPE) was included in a category representing milking equipment, given its use during the milking process. Each sample site category and the number of samples collected are described in Table 2. Prior to exploring detection results, the distribution of samples collected was described by sample characteristics. The number of samples in each site category collected in each state was reported, and the number of days from clinical signs (used to delineate outbreak start on each operation) to sample collection was compared by state and sample category using ANOVA with Tukey’s post hoc test (*p* < 0.05 as significant for comparisons).

Ct values represent an estimate of the amount of IAV (viral load) present in each sample and were reported by each NAHLN lab if values were less than the determined Ct cut-off of 40. Ct values from environmental samples with and without IAV detection were described by sample site category, state of sample collection, and the number of days from clinical signs to sample collection. For BTM, Ct values for each CO and CA farm were assigned based on the number of days between BTM collection and the date of first clinical signs in the herd. BTM Ct values across all farms were averaged over time using a rolling 3-day mean.

Because several factors may influence IAV presence in the environment, data were further explored via logistic regression in which the dependent variable was IAV detection. Results from sample site categories with >1 IAV detection were used in a mixed effect logistic model with state of collection and laboratory as random effects, and days from clinical signs to sample collection or sample site category as fixed effects. *p*-values were determined for mixed models using likelihood ratio tests comparing full to null models. All statistical analyses were completed using R version 2024.12.1+563 or later.

## 3. Results

A total of 94 (16.2%) of the 581 environmental samples collected in this investigation had IAV detected (Table 3 and Table S1). The largest percentage of positive samples were detected on parlor surfaces (28.9%) which had the second-largest number of samples collected for a single category (n = 121) and represented 20.8% of all samples collected. There were 26.6% of milking equipment and PPE samples that were positive, and they represented 30.5% of all samples collected. Meanwhile, 14.1% of wastewater/lagoon/manure samples were positive. Only a single sample from the housing category (an alleyway sample from MI), and one sample from the other category (a sample taken from equipment used to give cows liquid treatments in OH), were positive. This represents 1–2% of samples tested in each of those categories. No samples collected from the water tanks/feeders category (n = 62) had IAV detected. Of note, this category mainly comprised samples from waterers and mineral supplements, versus actual feed (Table S1). OH had the largest percentage of samples test positive at 27.1% (13/48) followed by CA at 21.6% (43/199). MI had 12.1% samples test positive (12/99) followed by CO with 11.1% (26/235).

The average Ct values from environmental samples with IAV detection were greater than 30 (Table 4), with parlor surfaces and milking equipment/PPE having the lowest Cts (avg. 31.9 ± 4.5 cycles and avg. 33.5 ± 4.3 cycles, respectively) compared to wastewater/lagoon/manure, housing and other sample site categories.

The number of samples collected from each site category varied by state (Figure S1). The days from clinical signs that samples were collected varied by sample site category, with wastewater/lagoon samples being collected later in a farm outbreak period compared to all other categories (*p* < 0.001 for all comparisons), and milking equipment/PPE samples being collected a greater number of days since initial clinical signs compared to samples taken from parlor surfaces (*p* < 0.001) and water tanks/feeders (*p* = 0.004) (Figure S2A). The days from clinical signs that samples were collected also varied by state, with CA samples, on average, being collected earlier in a farm outbreak period compared to other states, and MI samples, on average, being collected later in a farm outbreak period compared to other states (*p* < 0.001 for all comparisons; Figure S2B). For Michigan, sample collection ranged from 6 to 64 days after first clinical signs with a mean of 35 days. The Ohio farm had samples collected from 15 to 29 days after first clinical signs with a mean of 22 days. Colorado operations had samples collected 10 to 69 days after first clinical signs with a mean of 21 days. Lastly, California farms had samples collected 3 days prior to first clinical signs to 21 days after first clinical signs with a mean sampling time of 3 days after clinical signs.

**Figure 2:**
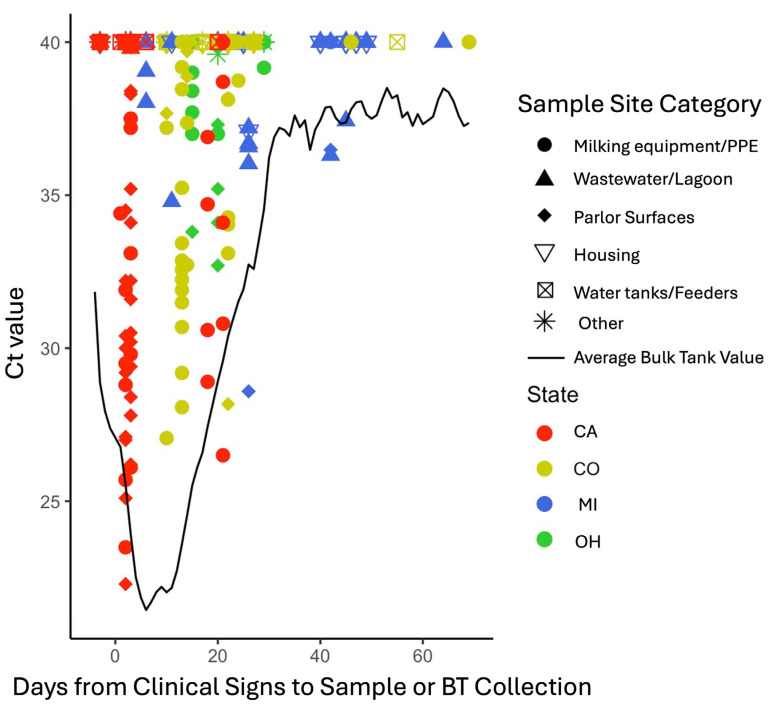
Ct values of environmental samples by state of collection and days from clinical signs to collection, overlaid with a curve of averaged bulk tank (BT) milk Ct values acquired from 10 enrolled herds during the period of sampling. Ct values < 40 from rRt-PCR testing were considered to have IAV detected, samples classified as negative have been given a value of 40.

BTM detections of IAV may represent the level of infection in each herd affected by H5N1. We performed a visual comparison of Ct values measured in both environmental and BTM samples, over the course of time after farm initial clinical signs. Samples with no detection were given a value of 40 and the distribution of Ct values was visualized by site category, state, and days after clinical signs that each sample was collected (Figure 2). In general, IAV detection and lower Ct values were seen from environmental samples collected during herd outbreak periods when average BTM Ct values were near or lower than 30, and Ct values from samples taken over time paralleled or were higher than average BTM Ct values used to designate herd viral burden (Figure 2). Data regarding BTM Ct values as they relate to other milk metrics (e.g., somatic cell count) was not available.

A total of 55 samples (8.8%) had indeterminate or quality flagged Ct results and were classified as negative. Environmental samples often contain matter that inhibits the rRT-PCR assay, which causes indeterminate results [22]. Indeterminate or quality flagged samples were collected in CO and MI and were from several sample site categories.

In addition to the sampling site, several factors may play a role in environmental IAV detections seen in this investigation, including the number of days that sampling occurred relative to the start of the outbreak (delineated by clinical signs) in each herd, the states samples were collected in, and the laboratories that tested samples. In this investigation, the state of collection also represents a different time of year, given when each state experienced H5N1-associated disease. Logistic modeling was used to understand the influence of these variables on detection results. The laboratories that tested samples were associated with the state of sample collection; two NAHLN laboratories tested samples from each state, and there was very little overlap between laboratories and states. The operations enrolled were also correlated with laboratory and state, as each dairy (with two exceptions) submitted samples to only one laboratory. Sample results from site categories with >1 IAV detection were categorized as 0/1 (non-detected/detected) and used as a dependent variable. Laboratory was nested within state as a random effect, and days from clinical signs to sample collection and sample site category were modeled as fixed effects. When compared to the null model, sample site category, when used as a fixed effect alone, was not associated with IAV detection (*p* = 0.246; Table 5). The number of days from first clinical signs that samples were collected, as a fixed effect alone, was associated with IAV detection (*p* = 0.0015; OR 0.96; 95% CI: 0.91–0.98), indicating that as the number of outbreak days a sample is collected increases, the odds of detecting IAV in that sample decreases (Table 5). When a mixed effects logistic regression model was created using both fixed variables of interest (sample site category, days from clinical signs that samples were collected), days from clinical signs was still associated with IAV detection and its effect estimate was relatively unchanged.

## 4. Discussion

**Figure 3:**
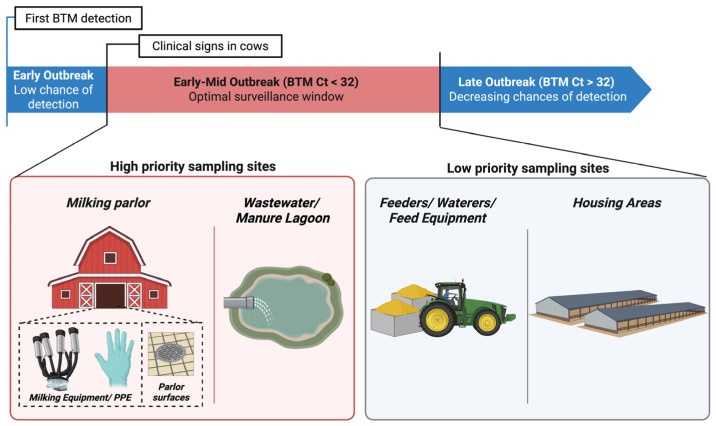
Summary diagram of study results, depicting a framework for continuing to investigate environmental sampling as a surveillance tool for IAV on dairies. Our results suggest that environmental samples collected very early in an outbreak and/or prior to herd clinical signs have low chances of IAV detection. Meanwhile, samples collected early to mid-outbreak and from specific sampling sites have the greatest chances for detection. Within these ‘high priority’ sites, the highest viral loads were recovered from milking equipment and parlor surface samples, making them the best choice if viral characterization is desired. Chances of environmental IAV detection decrease later in an outbreak period, as the BTM Ct value increases. Created in BioRender. Campbell, A. (2026). BioRender.com/aii71i2.

The current investigation describes environmental locations on 25 H5N1-affected dairy operations that had IAV viral RNA detection. Although differing facilities and changes in producer-allowed access resulted in variability in the number of samples and sample types collected on each operation, these results not only describe IAV detections on a large number of dairies for the first time, but they also provide a framework for future studies that assess dairy environmental sampling as an IAV surveillance tool (Figure 3).

Most IAV detections were made in samples collected from three site categories: milking equipment/PPE, parlor surfaces, and wastewater/lagoons. If individual cow or BTM sampling were not feasible on a farm and confirmation of H5N1 infection were needed, the information in this work can be used to target sampling of these specific sites. To the authors’ knowledge, only one other study has assessed contamination of the environment on a dairy farm experiencing H5N1, and although this study showed detections from different substrates (rubber, plastic), it did not provide information on specific locations or the number of days from first clinical signs that samples were collected [15]. Our results include samples taken from many operations at different outbreak time points, and suggest that IAV is more likely found in farm environments that have direct contact with milk. This hypothesis is supported by the relatively low Ct values from viral RNA (i.e., high viral load) detected in contaminated milk, although different cattle excretions containing viral RNA (e.g., urine, nasal discharge, blood) [2,23] may also contaminate these locations. It is also possible that the ‘micro’ environments with IAV detection (e.g., milking equipment, parlor surfaces, lagoons) are more conducive to IAV survival than the other locations tested. Studies have shown that IAV may persist better in environments with variable humidity, which are cool and away from sun exposure [24,25,26], although this may depend on viral subtype and attributes of the host shedding the virus [27,28,29]. Other factors that influence viral persistence include droplet size and evaporation rates, open versus closed air environments, and the porosity of surfaces IAV is deposited on [30,31]. Future studies should focus on understanding how these factors may influence IAV environmental persistence on dairies.

Our data show lower Ct values in milking equipment and parlor surface samples compared to lagoon samples. Although one may expect viral loads from wastewater streams to be lower compared to those of surfaces in the parlor due to dilution, the variability in timing of sample collection from different site categories precluded doing a direct comparison of these values, and Ct patterns in this work may reflect temporal dynamics rather than matrix effects alone. PCR detection does not differentiate between live virus and non-viable viral RNA, and virus isolation and whole genome sequencing (WGS) is often more successful in samples with higher viral loads [32,33]. The current investigation did not attempt to isolate live virus or perform WGS on collected samples, given both the time between sample collection and laboratory testing, and the magnitude of Ct values from many of the samples that had IAV detected. If future environmental sampling is done with a goal to perform virus isolation or WGS, targeting a location (e.g., parlor surface) identified as more likely to have a Ct < 30 should be pursued. Virus isolation rates from environmental samples collected during avian studies have been historically variable, likely due to the amount of viable virus in samples, sample processing and laboratory techniques [34,35,36,37,38]. Whether or not dairy environmental samples can support phylogenetic surveillance for H5N1 will hinge on future studies that assess both the viral load of collected samples, and the suitability of currently available viral culture techniques.

A relatively low percent (16.2%) of environmental samples had IAV detected in the current investigation. Sample collection methods and the distribution of samples available was influenced by the access and timeline of access to affected operations in each state (see Figure S2). Mixed effect logistic regression modeling that controlled for state and testing lab showed that IAV detection was influenced by the time from first clinical signs that samples were collected. This supports a notion that as an outbreak wanes and cattle recover from H5N1 disease, their viral shedding decreases, and this results in a decrease of virus in the environment. To understand how cattle shedding the virus influence environmental contamination, it would be advantageous to compare environmental detections of IAV to the prevalence of H5N1 infection in cows. However, we still lack a case definition for H5N1 disease in cows, which makes detection of disease variable among operations. Further, recent results have shown a high rate of H5N1 viral shedding in cows that lack clinical signs of disease [39]. For this investigation, BTM results from 10 of the enrolled dairies were used to represent the relative load of H5N1 in the dairy herd during the same outbreak time-periods that environmental samples were collected. The Ct values from both sample types increased over time in a similar fashion. Viral loads from environmental samples taken at similar time points were also near or lower than those of BTM, reinforcing the concept that excretions of cows shedding virus have a large impact on environmental IAV detection. These results and those of mixed modeling suggest that environmental sampling, if used for IAV detection, may be most successful during the middle or peak of a farm outbreak.

Environmental sampling of live bird markets has been used for early detection of H5N1 [38]. Because dairy environmental samples may be collected with ease compared to those from individual cattle, there is use in knowing if these samples could be used for early detection of H5N1-affected farms. One CA operation in the current investigation had environmental samples collected from multiple sites after BTM detection but prior to cattle showing clinical signs of disease. All environmental samples collected on this operation in this period did not have IAV detected. Although sampling this early was only performed on one operation, these results suggest that aggregate or BTM sampling may be superior for early detection of H5N1 affected dairy farms. This conclusion would be strengthened by additional environmental sampling on operations prior to clinical signs in cattle.

Importantly, it is unknown if the collection of other types of samples (e.g., bioaerosol samples or transient fomites like flies, peridomestic species, humans) from the early sampled operation and the other operations in this investigation would have yielded the same results [40]. Detection of IAV in air samples from porcine operations have been highly correlated with detection in environmental and individual animal samples [41]. During the 2024 dairy outbreak, IAV was unable to be detected in air samples collected on two H5N1 affected dairy operations in Texas [42]. However, detections were made in aerosols collected from affected dairy operations in California [43] and in a Vaccine and Infectious Disease Organization Containment Level 3 (VIDO CL3) large animal facility where cows were experimentally infected via the intramammary route [44]. Because shedding of the H5N1 virus into the air by experimentally infected ferrets has been correlated with transmissibility [45], future environmental work should also include the collection of bioaerosols from naturally infected herds. Prospective sampling of bioaerosols from naïve dairy herds in affected states may also be beneficial.

### Limitations

The current investigation had several limitations. The time between sample collection and laboratory testing did not allow for targeted repeat sampling of environmental sites with IAV detection. And although our data set was large and represented multiple operations, the number of samples from each site category collected from each operation differed, and total samples from each site category varied over the outbreak period. Although it is one of the only markers available, using the time from first clinical signs as a temporal indicator of when a farm outbreak starts has inherent limitations also, as clinical signs and producer detection thereof can vary among dairies. As mentioned in the discussion section, humidity, temperature, exposure to solar radiation, and other factors contribute to the persistence and viability of IAVs in the environment [24,25,26,46,47]. MI and OH, CO, and CA experienced H5N1 infection in dairy cattle during spring, summer, and fall, respectively, so sampling of operations in each of these states reflected weather patterns and seasonal variation in these parameters, along with variation in sampling strategies. Although the goal of the current analysis was to generally describe where IAV may be detected in the dairy environment to better inform environmental sampling as a dairy surveillance tool, future work should aim to quantify the variables that may influence the environmental presence of IAV. Future studies should also aim to consistently collect samples from sites prior to, during, and after herds experience clinical signs of disease. This would allow a temporal understanding of how the virus persists in certain sites on the dairy and, if appropriately powered, allow an assessment of which farm management factors influence the persistence of virus at these sites.

Since the H5N1 virus is a pathogen only recently impacting the dairy industry, we also do not have a great understanding of how sampling technique influences the detection sensitivity for virus in these environments. Methods used to sample poultry environments have been tested and refined since the 1970s [17], with studies showing that different sample pre-processing, elution, and concentration methods improve IAV detection in different scenarios [33,48,49]. The presence of inhibitors or other sample attributes may have affected IAV detection sensitivity, causing some of the indeterminate results seen in this investigation. Future work should explore how different sampling methods, especially the use of pre-wetted swabs [50], impact viral recovery and detection in samples taken from dairy surfaces.

## 5. Conclusions

Since detection in dairy cattle in early 2024, dairy surveillance for IAV has become a salient topic. The success of environmental sampling for IAV surveillance in any industry is dependent on whether the ultimate goals are detection of virus, phylogenetic analyses of viral strains, monitoring viral burden over time, or monitoring viral burden after initiating an intervention. Samples and results collected in this investigation provide a framework (Figure 3) within which to continue the assessment of environmental sampling as a surveillance tool to manage and mitigate dairy H5N1, especially as we learn more regarding viral shedding and transmission of the virus among dairy cattle.

## Figures and Tables

**Table 1:** Demographic characteristics of 25 dairy operations enrolled for environmental sampling, by State.

		MI		OH		CO		CA	
Characteristic	Level	Number	Percent	Number	Percent	Number	Percent	Number	Percent
Herd Size	<100	0	0.00	0	0.0	0	0.00	0	0.00
	100–499	3	25.0	0	0.00	0	0.00	0	0.00
	500+	9	75.0	1	100.0	6	100.0	6	100.0
Parlor Type	Herringbone	9	75.0	0	0.00	2	33.3	2	33.3
	Parallel	1	8.33	1	100.0	4	66.7	1	16.7
	Rotary	0	0.00	0	0.00	0	0.00	2	33.3
	Robotic	1	8.33	0	0.00	0	0.00	1	16.7
	NA ^1^	1	8.33	0	0.00	0	0.00	0	0.00
Breeds	Holstein ≥ 95%	10	83.3	0	0.00	3	50.0	5	83.3
	Jersey ≥ 95%	0	0.00	0	0.00	0	0.00	1	16.7
	Mixed	2	16.7	1	100.0	3	50.0	0	0.00
	**Total**	12	100.0	1	100.0	6	100.0	6	100.0

^1^ Refers to dairy with parlor at other site location.

**Table 2:** Summary of environmental samples for Influenza A virus detection by sample site category and source, including number of samples collected.

Sample Site Category	Sample Source	Total Samples Collected
Milking Equipment/PPE	Milking machine inflations	100
	Water hoses	25
	Gloves or apron of personnel	20
	Milking machines	9
	Dip equipment	8
	Milk lines/hoses	5
	Vacuum lines	4
	Bulk tank filter/hose	3
	Sinks	3
	Total	177
Parlor Surfaces	Surfaces	52
	Floor	38
	Drain	30
	Driveway next to parlor	1
	Total	121
Wastewater/lagoon/manure	Lagoon/Manure	39
	Lagoon Inflow	12
	Wastewater/Flush water	8
	Lagoon Outflow	5
	Manure Spreader	3
	Alley to Lagoon	2
	Holding tank/pit	2
	Total	71
Housing	Stanchion/Head lock	49
	Alleyway/Driveway	26
	Gates/Fences/Rails	12
	Free Stall/Other Surface	11
	Bedding	7
	Fan/Shade Support	3
	Barn Gutter	1
	Total	109
Water tanks/feeders	Waterers/tanks	45
	Feeding equipment	7
	Mineral block	5
	Feed Bunk/Feeders/Feed	5
	Total	62
Other	Farm equipment	21
	Surfaces in rooms for personnel	20
	Total	41
	**Total samples**	581

**Table 3:** Summary of environmental sample Influenza A virus rRt-PCR-positive test results, by sample site category and State of collection.

	PCR Positive Samples by State									
Sample Site Category	CA		CO		MI		OH		Category Total	
	Number	Percent	Number	Percent	Number	Percent	Number	Percent	Number	Percent
Milking Equipment/PPE	19/46	41.3%	21/106	19.8%	0/1	0.0%	7/24	29.2%	47/177	26.6%
Parlor Surfaces	23/60	38.3%	5/39	12.8%	2/6	33.3%	5/16	31.3%	35/121	28.9%
Wastewater/lagoon/manure	1/4	25.0%	0/2	0.0%	9/65	13.8%	0/0	0.0%	10/71	14.1%
Housing	0/44	0.0%	0/37	0.0%	1/26	3.8%	0/2	0.0%	1/109	0.9%
Water tanks/feeders	0/29	0.0%	0/30	0.0%	0/1	0.0%	0/2	0.0%	0/62	0.0%
Other	0/16	0.0%	0/21	0.0%	0/0	0.0%	1/4	25.0%	1/41	2.4%
**Total**	43/199	21.6%	26/235	11.1%	12/99	12.1%	13/48	27.1%	94/581	16.2%

**Table 4:** The average and range of Ct values measured in the five environmental sample site categories with Influenza A virus detected (Ct < 40) via rRt-PCR testing.

Sample Site Category	Average Positive Sample Ct Value (SD)	Range of Ct Values
Milking Equipment/PPE	33.5 (4.3)	23.5–39.9
Parlor Surfaces	31.9 (4.5)	22.3–39.7
Wastewater/lagoon/manure	37.2 (1.5)	34.8–39.8
Housing	37.1	37.1
Other	39.6	39.6

**Table 5:** Results of mixed effect logistic regression models evaluating the association between IAV detection and sample site category or days from clinical signs that samples were collected.

Variable	Level	Fixed Effects			Random Effects	
		Estimate (SE)	*p*-Value ^1^	Odds Ratio (CI)	Lab SD	State SD
Intercept		−1.62 (0.70)	0.246		1.08	0.00
Sample site category	Wastewater/Lagoon	Referent				
	Milking Equipment/PPE	0.85 (0.67)		2.32 (0.62–9.10)		
	Parlor Surfaces	1.06 (0.65)		2.88 (0.80–10.67)		
Intercept		0.148 (0.51)	0.0015		0.96	0.00
Days from clinical signs to sample collection		−0.052 (0.018)		0.95 (0.91–0.98)		

^1^ Maximum likelihood estimation comparing specified model to a null model using only random effects.

## Data Availability

The original contributions presented in this study are included in the article/Supplementary Materials. Further inquiries can be directed to the corresponding author.

## Supplementary Materials

The following supporting information can be downloaded at: https://www.mdpi.com/article/10.3390/microorganisms14030584/s1, Figure S1: Number of environmental samples collected by sample site category and by State for Influenza A virus detection; Figure S2: (A) Boxplot of the days from clinical signs to environmental sample collection for Influenza A detection by sample site category. (B) Boxplot of the days from clinical signs of Influenza A to environmental sample collection by state of collection. Statistical significance determined using ANOVA with Tukey’s post-hoc test: * *p* < 0.05, ** *p* < 0.01, *** *p* < 0.001; Table S1: rRT-PCR results and specific location data associated with collected study samples.

## Abbreviations

The following abbreviations are used in this manuscript:

IAV	Influenza A virus
HPAI	Highly pathogenic avian influenza
BTM	Bulk tank milk
CA	California
CO	Colorado
MI	Michigan
OH	Ohio
MDARD	Michigan Department of Agriculture and Rural Development
ODA	Ohio Department of Agriculture
USDA:APHIS:VS	United States Department of Agriculture’s Animal and Plant Health Inspection Service Veterinary Services
CDA	Colorado Department of Agriculture
CSU	Colorado State University
rRT-PCR	Real-time reverse transcription polymerase chain reaction
CDFA	California Department of Food and Agriculture
LVC	Lander Veterinary Clinic
NVSL	National Veterinary Services Laboratories
UVT	Universal transport media
BHI	Brain heart infusion
MTM	Molecular transport media
NAHLN	National animal health laboratory network
Ct	Cycle threshold
WGS	Whole genome sequencing
PPE	Personal protective equipment

## References

[1] Burrough E.R., Magstadt D.R., Petersen B., Timmermans S.J., Gauger P.C., Zhang J., Siepker C., Mainenti M., Li G., Thompson A.C. (2024). Highly Pathogenic Avian Influenza A(H5N1) Clade 2.3.4.4b Virus Infection in Domestic Dairy Cattle and Cats, United States, 2024. Emerg. Infect. Dis..

[2] Caserta L.C., Frye E.A., Butt S.L., Laverack M.A., Nooruzzaman M., Covalenda L.M., Thompson A., Prarat Koscielny M., Cronk B., Johnson A. (2024). From Birds to Mammals: Spillover of Highly Pathogenic Avian Influenza H5N1 Virus to Dairy Cattle Led to Efficient Intra- and Interspecies Transmission. bioRxiv.

[3] Nguyen T.-Q., Hutter C.R., Markin A., Thomas M., Lantz K., Killian M.L., Janzen G.M., Vijendran S., Wagle S., Inderski B. (2025). Emergence and Interstate Spread of Highly Pathogenic Avian Influenza A(H5N1) in Dairy Cattle in the United States. Science.

[4] CDC H5 Bird Flu: Current Situation. https://www.cdc.gov/bird-flu/situation-summary/index.html.

[5] Animal and Plant Health Inspection Service HPAI Confirmed Cases in Livestock. https://www.aphis.usda.gov/livestock-poultry-disease/avian/avian-influenza/hpai-detections/hpai-confirmed-cases-livestock.

[6] Animal and Plant Health Inspection Service APHIS Confirms D1.1 Genotype in Dairy Cattle in Nevada. https://www.aphis.usda.gov/news/program-update/aphis-confirms-d11-genotype-dairy-cattle-nevada-0.

[7] Lombard J., Stenkamp-Strahm C., McCluskey B., Abdul-Hamid C., Cardona C., Petersen B., Russo K. (2025). The One Health Challenges and Opportunities of the H5N1 Outbreak in Dairy Cattle in the United States. J. Dairy Sci..

[8] Dairy Farm Biosecurity: Preventing the Spread of H5N1. https://www.aphis.usda.gov/sites/default/files/dairy-cattle-biosecurity-measures.pdf.

[9] USDA Announces New Federal Order, Begins National Milk Testing Strategy to Address H5N1 in Dairy Herds. https://www.usda.gov/article/usda-announces-new-federal-order-begins-national-milk-testing-strategy-address-h5n1-dairy-herds.

[10] Sutton M., Falender R., Scholz R., Mickle D., Cieslak P., Phatak G., Radniecki T. (2025). Avian Influenza A (H5) in Wastewater, July 2024 to February 2025. JAMA Netw. Open.

[11] Tiwari A., Patnayak D.P., Chander Y., Parsad M., Goyal S.M. (2006). Survival of Two Avian Respiratory Viruses on Porous and Nonporous Surfaces. Avian Dis..

[12] Wood J.P., Choi Y.W., Chappie D.J., Rogers J.V., Kaye J.Z. (2010). Environmental Persistence of a Highly Pathogenic Avian Influenza (H5N1) Virus. Environ. Sci. Technol..

[13] Torremorell M., Alonso C., Davies P.R., Raynor P.C., Patnayak D., Torchetti M., McCluskey B. (2016). Investigation into the Airborne Dissemination of H5N2 Highly Pathogenic Avian Influenza Virus During the 2015 Spring Outbreaks in the Midwestern United States. Avian Dis..

[14] Lopez K.M., Nezworski J., Rendahl A., Culhane M., Flores-Figueroa C., Muñoz-Aguayo J., Halvorson D.A., Johnson R., Goldsmith T., Cardona C.J. (2018). Environmental Sampling Survey of H5N2 Highly Pathogenic Avian Influenza–Infected Layer Chicken Farms in Minnesota and Iowa. Avian Dis..

[15] Singh G., Trujillo J.D., McDowell C.D., Matias-Ferreyra F., Kafle S., Kwon T., Gaudreault N.N., Fitz I., Noll L., Morozov I. (2024). Detection and Characterization of H5N1 HPAIV in Environmental Samples from a Dairy Farm. Virus Genes.

[16] Le Sage V., Campbell A.J., Reed D.S., Duprex W.P., Lakdawala S.S. (2024). Persistence of Influenza H5N1 and H1N1 Viruses in Unpasteurized Milk on Milking Unit Surfaces. Emerg. Infect. Dis..

[17] Hood G., Roche X., Brioudes A., Von Dobschuetz S., Fasina F.O., Kalpravidh W., Makonnen Y., Lubroth J., Sims L. (2021). A Literature Review of the Use of Environmental Sampling in the Surveillance of Avian Influenza Viruses. Transbound. Emerg. Dis..

[18] Stadler J., Zwickl S., Gumbert S., Ritzmann M., Lillie-Jaschniski K., Harder T., Graaf-Rau A., Skampardonis V., Eddicks M. (2024). Influenza Surveillance in Pigs: Balancing Act between Broad Diagnostic Coverage and Specific Virus Characterization. Porc. Health Manag..

[19] Garrido-Mantilla J., Alvarez J., Culhane M., Nirmala J., Cano J.P., Torremorell M. (2019). Comparison of Individual, Group and Environmental Sampling Strategies to Conduct Influenza Surveillance in Pigs. BMC Vet. Res..

[20] 2024 Highly Pathogenic Avian Influenza (H5N1)—Michigan Dairy Herd and Poultry Flock Summary. https://www.aphis.usda.gov/sites/default/files/hpai-h5n1-dairy-cattle-mi-epi-invest.pdf.

[21] Department of Agriculture Colorado State Veterinarian Now Requiring HPAI Testing of Commercial Dairy Cow Operations. https://ag.colorado.gov/press-release/colorado-state-veterinarian-now-requiring-hpai-testing-of-commercial-dairy-cow.

[22] Schrader C., Schielke A., Ellerbroek L., Johne R. (2012). PCR Inhibitors—Occurrence, Properties and Removal. J. Appl. Microbiol..

[23] Lombard J., Stenkamp-Strahm C., McCluskey B., Melody B. (2025). Evidence of Viremia in Dairy Cows Naturally Infected with Influenza A Virus, California, USA. Emerg. Infect. Dis..

[24] Brown J.D., Swayne D.E., Cooper R.J., Burns R.E., Stallknecht D.E. (2007). Persistence of H5 and H7 Avian Influenza Viruses in Water. Avian Dis..

[25] Sagripanti J.-L., Lytle C.D. (2007). Inactivation of Influenza Virus by Solar Radiation. Photochem. Photobiol..

[26] Lowen A.C., Mubareka S., Steel J., Palese P. (2007). Influenza Virus Transmission Is Dependent on Relative Humidity and Temperature. PLoS Pathog..

[27] Kormuth K.A., Lin K., Prussin A.J., Vejerano E.P., Tiwari A.J., Cox S.S., Myerburg M.M., Lakdawala S.S., Marr L.C. (2018). Influenza Virus Infectivity Is Retained in Aerosols and Droplets Independent of Relative Humidity. J. Infect. Dis..

[28] Kormuth K.A., Lin K., Qian Z., Myerburg M.M., Marr L.C., Lakdawala S.S. (2019). Environmental Persistence of Influenza Viruses Is Dependent upon Virus Type and Host Origin. mSphere.

[29] Qian Z., Morris D.H., Avery A., Kormuth K.A., Le Sage V., Myerburg M.M., Lloyd-Smith J.O., Marr L.C., Lakdawala S.S. (2023). Variability in Donor Lung Culture and Relative Humidity Impact the Stability of 2009 Pandemic H1N1 Influenza Virus on Nonporous Surfaces. Appl. Environ. Microbiol..

[30] French A.J., Longest A.K., Pan J., Vikesland P.J., Duggal N.K., Marr L.C., Lakdawala S.S. (2023). Environmental Stability of Enveloped Viruses Is Impacted by Initial Volume and Evaporation Kinetics of Droplets. mBio.

[31] Weber T.P., Stilianakis N.I. (2008). Inactivation of Influenza A Viruses in the Environment and Modes of Transmission: A Critical Review. J. Infect..

[32] Latorre-Margalef N., Avril A., Tolf C., Olsen B., Waldenström J. (2016). How Does Sampling Methodology Influence Molecular Detection and Isolation Success in Influenza A Virus Field Studies?. Appl. Environ. Microbiol..

[33] Horm S.V., Deboosere N., Gutiérrez R.A., Vialette M., Buchy P. (2011). Direct Detection of Highly Pathogenic Avian Influenza A/H5N1 Virus from Mud Specimens. J. Virol. Methods.

[34] Haynes L., Arzey E., Bell C., Buchanan N., Burgess G., Cronan V., Dickason C., Field H., Gibbs S., Hansbro P. (2009). Australian Surveillance for Avian Influenza Viruses in Wild Birds between July 2005 and June 2007. Aust. Vet. J..

[35] Onuma M., Kakogawa M., Yanagisawa M., Haga A., Okano T., Neagari Y., Okano T., Goka K., Asakawa M. (2017). Characterizing the Temporal Patterns of Avian Influenza Virus Introduction into Japan by Migratory Birds. J. Vet. Med. Sci..

[36] Vong S., Ly S., Mardy S., Holl D., Buchy P. (2008). Environmental Contamination during Influenza A Virus (H5N1) Outbreaks, Cambodia, 2006. Emerg. Infect. Dis. J..

[37] Grillo V., Arzey K., Hansbro P., Hurt A., Warner S., Bergfeld J., Burgess G., Cookson B., Dickason C., Ferenczi M. (2015). Avian Influenza in Australia: A Summary of 5 Years of Wild Bird Surveillance. Aust. Vet. J..

[38] Cauthen A.N., Swayne D.E., Schultz-Cherry S., Perdue M.L., Suarez D.L. (2000). Continued Circulation in China of Highly Pathogenic Avian Influenza Viruses Encoding the Hemagglutinin Gene Associated with the 1997 H5N1 Outbreak in Poultry and Humans. J. Virol..

[39] Stenkamp-Strahm C.S., Lombard J., Melody B., Brinson P., McCluskey B. (2025). Influenza A Virus detection in Bulk Tank and Pen Level Milk from Dairies Affected by Highly Pathogenic Avian Influenza H5N1. medRxiv.

[40] Nayduch D., Scroggs S.L., Shults P., Brendel L.A., Resiter-Hendricks L.M., Taylor C., Bird E., Lopez B., Marshall E.S. (2026). Detection of H5N1 highly pathogenic avian influenza virus RNA in filth flies collected from California farms in 2024. Sci. Rep..

[41] Prost K., Kloeze H., Mukhi S., Bozek K., Poljak Z., Mubareka S. (2019). Bioaerosol and Surface Sampling for the Surveillance of Influenza A Virus in Swine. Transbound. Emerg. Dis..

[42] Shittu I., Silva D., Oguzie J.U., Marushchak L.V., Olinger G.G., Lednicky J.A., Trujillo-Vargas C.M., Schneider N.E., Hao H., Gray G.C. (2024). A One Health Investigation into H5N1 Avian Influenza Virus Epizootics on Two Dairy Farms. Clin. Infect. Dis..

[43] Campbell A.J., Shephard M., Paulos A.P., Pauly M., Vu M., Stenkamp-Strahm C., Bushfield K., Hunter-Binns B., Sablon O., Bendall E.E. (2025). Surveillance on California Dairy Farms Reveals Multiple Sources of H5N1 Transmission. bioRxiv.

[44] Facciuolo A., Aubrey L., Barron-Castillo U., Berube N., Norleen C., McCreary S., Huang Y., Pessoa N., Jacome L.M., Mubareka S. (2025). Dairy Cows Develop Protective Immunity against Reinfection with Bovine H5N1 Influenza Virus. Nat. Microbiol..

[45] Tosheva I.I., Filaire F., Rijnink W.F., De Meulder D., Van Kekem B., Bestebroer T.M., Funk M., Spronken M.I., Cáceres C.J., Perez D.R. (2024). Influenza A(H5N1) Shedding in Air Corresponds to Transmissibility in Mammals. Nat. Microbiol..

[46] Keeler S.P., Dalton M.S., Cressler A.M., Berghaus R.D., Stallknecht D.E. (2024). Abiotic factors affecting the persistence of avian influenza virus in surface waters of waterfowl habitats. Appl. Environ. Microbiol..

[47] Brown J.D., Goekjian G., Poulson R., Valeika S., Stallknecht D.E. (2009). Avian Influenza Virus in Water: Infectivity Is Dependent on pH, Salinity and Temperature. Vet. Microbiol..

[48] Deboosere N., Horm S.V., Pinon A., Gachet J., Coldefy C., Buchy P., Vialette M. (2011). Development and Validation of a Concentration Method for the Detection of Influenza A Viruses from Large Volumes of Surface Water. Appl. Environ. Microbiol..

[49] Rönnqvist M., Ziegler T., von Bonsdorff C.-H., Maunula L. (2012). Detection Method for Avian Influenza Viruses in Water. Food Environ. Virol..

[50] Julian T.R., Tamayo F.J., Leckie J.O., Boehm A.B. (2011). Comparison of Surface Sampling Methods for Virus Recovery from Fomites. Appl. Environ. Microbiol..

