# Supplementary material for: Dairy Sites with Milk Exposure Are Most Likely to Have Detection of Influenza A Virus
Source: Microorganisms. 2026 Mar 5;14(3):584. doi: 10.3390/microorganisms14030584 (PMC13028609; doi:10.3390/microorganisms14030584)
Supplement: Supplementary file 1 [file microorganisms-14-00584-s001.zip › microorganisms-4074718-supplementary.pdf]

SUPPLEMENTAL TABLES AND FIGURES

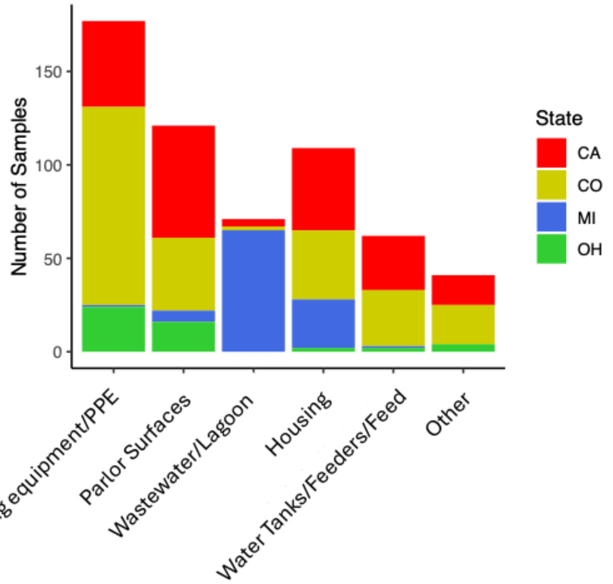

Figure S1: Number of environmental samples collected by sample site category and by State for Influenza A virus detection.

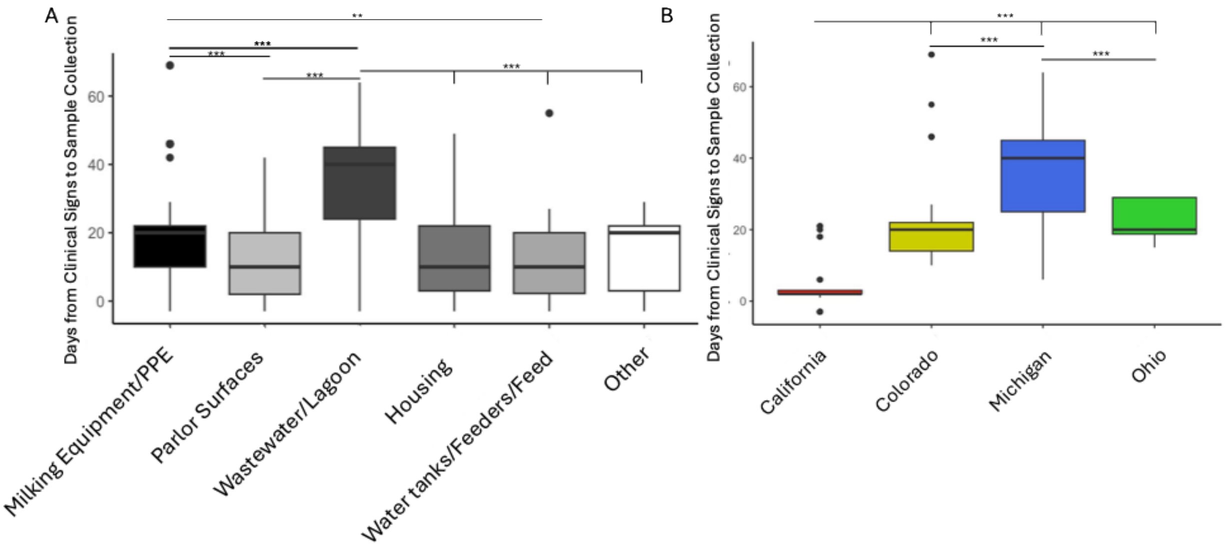

Figure S2: Panel A: Boxplot of the days from clinical signs to environmental sample collection for Influenza A detection by sample site category. Panel B: Boxplot of the days from clinical signs of Influenza A to environmental sample collection by state of collection. Statistical significance determined using ANOVA with Tukey's post-hoc test: * $p < 0.05$, ** $p < 0.01$, *** $p < 0.001$.

Table S1. rRT-PCR results and specific location data associated with collected study samples

Collection date	State	Location	Ct value[1]
4/5/24	OH	Inflation/teat liner	38.4
4/5/24	OH	Inflation/teat liner	ND
4/5/24	OH	Inflation/teat liner	ND
4/5/24	OH	Inflation/teat liner	ND
4/5/24	OH	Gloves	ND
4/5/24	OH	Gloves	37
4/5/24	OH	Teat dip hose	37.7
4/5/24	OH	Water hose	39
4/5/24	OH	Stall dividers	33.8
4/5/24	OH	Stall dividers	ND
4/5/24	OH	Stall dividers	ND
4/5/24	OH	Stall dividers	ND
4/10/24	OH	Floor	35.2
4/10/24	OH	Floor	32.7
4/10/24	OH	Drain	34.1
4/10/24	OH	Drain	37.3
4/10/24	OH	Parlor surface	ND
4/10/24	OH	Parlor surface	ND
4/10/24	OH	Gloves	ND
4/10/24	OH	Water drop hose	39.9
4/10/24	OH	Gloves	ND
4/10/24	OH	Water drop hose	ND
4/10/24	OH	Inflation/teat liner	ND
4/10/24	OH	Inflation/teat liner	ND
4/10/24	OH	Teat dip cups	ND
4/10/24	OH	Teat dip cups	ND
4/10/24	OH	Bedding	ND
4/10/24	OH	Waterer	ND
4/10/24	OH	Bedding	ND
4/10/24	OH	Waterer	ND
4/10/24	OH	Drench equip	39.6
4/10/24	OH	Drench equip	ND
4/19/24	OH	Inflation/teat liner	ND
4/19/24	OH	Inflation/teat liner	ND
4/19/24	OH	Teat dip cups	ND
4/19/24	OH	Teat dip cups	ND
4/19/24	OH	Gloves	ND
4/19/24	OH	Gloves	ND
4/19/24	OH	Drop hoses	ND
4/19/24	OH	Drop hoses	39.16
4/19/24	OH	Surface	ND
4/19/24	OH	Surface	ND
4/19/24	OH	Floor	ND
4/19/24	OH	Floor	ND
4/19/24	OH	Drain	ND
4/19/24	OH	Drain	ND
4/19/24	OH	Washing machine	ND
4/19/24	OH	Washing machine	ND
6/27/24	CO	Parlor restroom	ND
6/27/24	CO	Parlor office	ND

6/27/24	CO	Parlor break room	ND
6/27/24	CO	Parlor door handles	ND
6/27/24	CO	Inflation/teat liner	ND
6/27/24	CO	Gloves	ND
6/27/24	CO	Teat dip hose	ND
6/27/24	CO	Drain	ND
6/27/24	CO	Water hose	ND
6/27/24	CO	Floor stanchions	ND
6/27/24	CO	Bulk Tank Ladder	ND
6/27/24	CO	Bulk Tank Sink	ND
6/27/24	CO	Bulk Tank Filters	27.06
6/27/24	CO	Bulk Tank Hoses	37.2
6/27/24	CO	Exterior of Pasturizer	ND
6/27/24	CO	Sink	ND
6/27/24	CO	Doors	ND
6/27/24	CO	Milk claw / Hose	ND
6/27/24	CO	Parlor Floor Grates	37.67
6/27/24	CO	Parlor Control Buttons	ND
6/27/24	CO	Parlor Holding Pen	ND
6/27/24	CO	Treatment pen water tanks	ND
6/27/24	CO	Hoof Table	ND
6/27/24	CO	Treatment Pen Head Catches	ND
6/27/24	CO	Water tanks	ND
6/27/24	CO	Free Stalls	ND
6/27/24	CO	Treatment Room	ND
6/27/24	CO	Push Up Tractor	ND
6/27/24	CO	Lock-ups	ND
6/27/24	CO	Water Tanks	ND
6/27/24	CO	Sick Pen Lock-up Rail	ND
6/27/24	CO	Sick Pen water tanks	ND
6/27/24	CO	Holding Pen	ND
6/27/24	CO	Sick Pen Side Rail	ND
6/27/24	CO	Walk Up Alley To Parlor	ND
6/27/24	CO	Treatment Room	ND
6/27/24	CO	Treatment Chute	ND
6/27/24	CO	Close-Up Water Tank	ND
7/10/24	MI	Alley	ND
7/10/24	MI	Alley	ND
7/10/24	MI	Alley	ND
7/10/24	MI	Alley	ND
7/10/24	MI	Alley	ND
7/10/24	MI	Alley	ND
7/10/24	MI	Lagoon	ND
7/10/24	MI	Lagoon	ND
7/10/24	MI	Lagoon	ND
7/10/24	MI	Lagoon	ND
7/10/24	MI	Lagoon	ND
7/10/24	MI	Lagoon	ND
7/10/24	MI	Lagoon	ND
7/10/24	MI	Lagoon	ND
7/10/24	MI	Lagoon	ND
6/6/24	MI	Lagoon inflow	36.3
6/6/24	MI	Milk house floor	ND

6/6/24	MI	Lagoon outflow	ND
6/6/24	MI	Drain	ND
6/6/24	MI	Inflation/teat liner	ND
6/6/24	MI	Driveway	36.48
5/18/24	MI	Manure	ND
5/18/24	MI	Manure	ND
5/21/24	MI	Manure spreader	39.05
5/21/24	MI	Manure spreader	ND
5/21/24	MI	Pond manure	ND
5/21/24	MI	Manure spreader start	38.03
6/6/24	MI	Holding Pen	ND
6/6/24	MI	Lagoon inlet	ND
6/6/24	MI	Lagoon inlet/outlet	ND
6/11/24	MI	Lagoon inlet	36.58
6/11/24	MI	Lagoon inlet	36.03
6/11/24	MI	Lagoon outflow	36.69
6/11/24	MI	Lagoon outflow	37.2
6/11/24	MI	Drain	28.59
6/11/24	MI	6XALLEYWAY	37.1
6/11/24	MI	Lagoon inflow	ND
6/11/24	MI	Lagoon inflow	ND
6/11/24	MI	Lagoon outflow	ND
6/11/24	MI	Lagoon outflow	ND
6/11/24	MI	Drain	ND
6/11/24	MI	Holding tank inlet	ND
6/11/24	MI	Holding tank inlet	ND
6/11/24	MI	Barn gutter	ND
6/11/24	MI	Lagoon	34.79
6/11/24	MI	Lagoon	ND
6/11/24	MI	Drain	ND
6/11/24	MI	Reception pit	ND
6/14/24	MI	Barn alley	ND
6/14/24	MI	Barn alley	ND
6/14/24	MI	Barn alley	ND
6/14/24	MI	Barn alley	ND
6/14/24	MI	Driveway	ND
6/14/24	MI	Hospital pen allwy	ND
6/14/24	MI	Lagoon	ND
6/14/24	MI	Lagoon back	ND
6/14/24	MI	Lagoon front	ND
6/14/24	MI	Lagoon inflow pipe	ND
6/14/24	MI	Lagoon inflow	ND
6/14/24	MI	LAGOON2	ND
6/14/24	MI	Lagoon inflow	ND
6/14/24	MI	Whey	ND
6/20/24	MI	Alley to lagoon	ND
6/20/24	MI	Alley to lagoon	ND
6/20/24	MI	Barn alley	ND
6/20/24	MI	Barn alley	ND
6/20/24	MI	Lagoon end	ND
6/20/24	MI	Lagoon inflow	ND
6/20/24	MI	Lagoon inflow	ND
6/20/24	MI	Lagoon middle	ND

6/20/24	MI	Parlor wastewater	ND
6/25/24	MI	Alley	ND
6/25/24	MI	Alley	ND
6/25/24	MI	Alley	ND
6/25/24	MI	Alley	ND
6/25/24	MI	Lagoon	ND
6/25/24	MI	Lagoon	ND
6/25/24	MI	Lagoon	ND
6/25/24	MI	Lagoon	ND
7/16/24	MI	Lagoon	ND
6/27/24	MI	Alley	ND
6/27/24	MI	Alley	ND
6/27/24	MI	Alley	ND
6/27/24	MI	Alley	ND
6/27/24	MI	Alley	ND
6/27/24	MI	Alley	ND
6/27/24	MI	Lagoon/wastewater	ND
6/27/24	MI	Lagoon/wastewater	ND
6/27/24	MI	Lagoon/wastewater	ND
6/27/24	MI	Lagoon	ND
6/27/24	MI	Lagoon	ND
6/27/24	MI	Lagoon	ND
6/27/24	MI	Lagoon	ND
6/27/24	MI	Lagoon	ND
6/27/24	MI	Lagoon	ND
6/27/24	MI	Lagoon	ND
6/27/24	MI	Lagoon	37.43
6/27/24	MI	Lagoon	ND
6/27/24	MI	Lagoon	ND
10/29/24	CA	Inflation/teat liner	ND
10/29/24	CA	Drain	ND
10/29/24	CA	Feed push up tractor	ND
10/29/24	CA	Waterer	ND
10/29/24	CA	Stanchion	ND
10/29/24	CA	Waterer	ND
10/29/24	CA	Stanchion	ND
10/29/24	CA	Stanchion	ND
10/29/24	CA	Waterer	ND
10/29/24	CA	Free stall fan	ND
10/29/24	CA	Equipment	ND
10/29/24	CA	Inflation/teat liner	ND
10/29/24	CA	Flush water	ND
10/29/24	CA	Flush water	ND
10/29/24	CA	Mineral block	ND
10/29/24	CA	Equipment	ND
10/29/24	CA	Stanchion	ND
10/29/24	CA	Waterer	ND
10/29/24	CA	Waterer	ND
10/29/24	CA	Parlor floor	ND
10/29/24	CA	Parlor floor	ND
10/29/24	CA	Parlor surface	ND
10/29/24	CA	Parlor surface	ND
10/29/24	CA	Parlor surface	ND

10/29/24	CA	Parlor surface	ND
10/29/24	CA	Inflation/teat liner	ND
11/7/24	CA	Waterer	ND
11/7/24	CA	Waterer	ND
11/7/24	CA	Waterer	ND
11/7/24	CA	Waterer	ND
12/2/24	CA	Inflation/teat liner	26.1
12/2/24	CA	Inflation/teat liner	ND
12/2/24	CA	Inflation/teat liner	ND
12/2/24	CA	Inflation/teat liner	ND
12/2/24	CA	Vacuum Lines	ND
12/2/24	CA	Vacuum Lines	ND
12/2/24	CA	Vacuum Lines	33.1
12/2/24	CA	Vacuum Lines	ND
12/2/24	CA	Parlor Drain	27.8
12/2/24	CA	Parlor Drain	29.4
12/2/24	CA	Parlor Drain	26.2
12/2/24	CA	Parlor Drain	28.4
12/2/24	CA	Parlor Floor	30.2
12/2/24	CA	Parlor Floor	32.2
12/2/24	CA	Parlor Floor	ND
12/2/24	CA	Parlor Floor	ND
12/2/24	CA	Gloves	37.5
12/2/24	CA	Gloves	ND
12/2/24	CA	Parlor Surfaces	ND
12/2/24	CA	Parlor Surfaces	ND
12/2/24	CA	Equipment	ND
12/2/24	CA	Equipment	ND
12/2/24	CA	Equipment	ND
12/2/24	CA	Equipment	ND
12/2/24	CA	Waterer	ND
12/2/24	CA	Commodity Shed	ND
12/2/24	CA	Equipment	ND
12/2/24	CA	Stanchion	ND
12/2/24	CA	Stanchion	ND
12/2/24	CA	Stanchion	ND
12/2/24	CA	Stanchion	ND
12/2/24	CA	Waterer	ND
12/2/24	CA	Waterer	ND
12/2/24	CA	Mineral Blocks	ND
12/2/24	CA	Free Stall	ND
12/2/24	CA	Stanchion	ND
12/2/24	CA	Stanchion	ND
12/2/24	CA	Free Stall	ND
12/2/24	CA	Free Stall	ND
12/2/24	CA	Stanchion	ND
12/2/24	CA	Milking Parlor	ND
12/2/24	CA	Milking Parlor	ND
11/30/24	CA	Hosp milk hose	34.4
11/30/24	CA	Waterer	ND
12/3/24	CA	Parlor floor	ND
12/3/24	CA	Parlor floor	ND
12/3/24	CA	Parlor floor	ND

12/3/24	CA	Parlor floor	ND
12/3/24	CA	Milking machines	ND
12/3/24	CA	Milking machines	ND
12/3/24	CA	Milking machines	ND
12/3/24	CA	Milking machines	ND
12/3/24	CA	Parlor surfaces	ND
12/3/24	CA	Parlor surfaces	ND
12/3/24	CA	Parlor surfaces	ND
12/3/24	CA	Parlor surfaces	ND
12/3/24	CA	Parlor drain	ND
12/3/24	CA	Parlor drain	ND
12/3/24	CA	Parlor drain	ND
12/3/24	CA	Parlor drain	ND
12/3/24	CA	Parlor surfaces	ND
12/3/24	CA	Gloves	ND
12/3/24	CA	Water drop hoses	ND
12/3/24	CA	Water drop hoses	ND
12/3/24	CA	Stanchions	ND
12/3/24	CA	Stanchions	ND
12/3/24	CA	Stanchions	ND
12/3/24	CA	Stanchions	ND
12/3/24	CA	Equipment	ND
12/3/24	CA	Equipment	ND
12/3/24	CA	Equipment	ND
12/3/24	CA	Equipment	ND
12/3/24	CA	Waterer	ND
12/3/24	CA	Waterer	ND
12/3/24	CA	Waterer	ND
12/3/24	CA	Hospital stanchions	ND
12/3/24	CA	Hospital stanchions	ND
12/3/24	CA	Hospital stanchions	ND
12/3/24	CA	Waterer	ND
12/3/24	CA	Gates	ND
12/3/24	CA	Gates	ND
12/3/24	CA	Stanchions	ND
12/3/24	CA	Stanchions	ND
12/3/24	CA	Stanchions	ND
12/3/24	CA	Milking parlor	ND
12/3/24	CA	Milking parlor	ND
12/3/24	CA	Milking parlor	ND
12/4/24	CA	Teat cup liners	ND
12/4/24	CA	Teat cup liners	ND
12/4/24	CA	Teat cup liners	ND
12/4/24	CA	Milking unit	ND
12/4/24	CA	Parlor floor	ND
12/4/24	CA	Parlor floor	30.5
12/4/24	CA	Parlor floor	ND
12/4/24	CA	Parlor drain	35.2
12/4/24	CA	Parlor drain	ND
12/4/24	CA	Parlor drain	ND
12/4/24	CA	Parlor surfaces	38.3
12/4/24	CA	Parlor surfaces	34.1
12/4/24	CA	Parlor surfaces	ND

12/4/24	CA	Parlor surfaces	38.4
12/4/24	CA	Water drop hoses	37.2
12/4/24	CA	Water drop hoses	ND
12/4/24	CA	Milker gloves	ND
12/4/24	CA	Milker apron	29.8
12/4/24	CA	Parlor surfaces	31.6
12/4/24	CA	Parlor surfaces	ND
12/4/24	CA	Waterer	ND
12/4/24	CA	Gates	ND
12/4/24	CA	Stanchion	ND
12/4/24	CA	Stanchion	ND
12/4/24	CA	Waterer	ND
12/4/24	CA	Stanchion	ND
12/4/24	CA	Stanchion	ND
12/4/24	CA	Flush water	39.8
12/4/24	CA	Flush water	ND
12/4/24	CA	Drenching equipment	ND
12/4/24	CA	Equipment	ND
12/4/24	CA	Equipment	ND
12/4/24	CA	Gates	ND
12/4/24	CA	Gates	ND
12/4/24	CA	Gates	ND
12/2/24	CA	Inflation/teat liner	23.5
12/2/24	CA	Inflation/teat liner	31.9
12/2/24	CA	Inflation/teat liner	28.8
12/2/24	CA	Inflation/teat liner	29.5
12/2/24	CA	Parlor Floor	30
12/2/24	CA	Parlor Floor	29.2
12/2/24	CA	Parlor Floor	27.1
12/2/24	CA	Parlor Drain	32.2
12/2/24	CA	Parlor Drain	25.7
12/2/24	CA	Parlor Drain	29.5
12/2/24	CA	Parlor Surfaces	ND
12/2/24	CA	Parlor Surfaces	22.3
12/2/24	CA	Parlor Surfaces	27
12/2/24	CA	Parlor Surfaces	25.1
12/2/24	CA	Milker Gloves	ND
12/2/24	CA	Water Drop Hoses	ND
12/2/24	CA	Gloves	25.7
12/2/24	CA	Parlor Floor	34.5
12/2/24	CA	Parlor Floor	ND
12/2/24	CA	Parlor Floor	30.4
12/2/24	CA	Equipment	ND
12/2/24	CA	Waterer	ND
12/2/24	CA	Waterer	ND
12/2/24	CA	Equipment - feed robot	ND
12/2/24	CA	Stanchion	ND
12/2/24	CA	Stanchion	ND
12/2/24	CA	Stanchion	ND
12/2/24	CA	Waterer	ND
12/2/24	CA	Free Stall	ND
12/2/24	CA	Free Stall	ND
12/2/24	CA	Equipment	ND

12/2/24	CA	Free Stall	ND
12/2/24	CA	Free Stall	ND
12/2/24	CA	Stanchion	ND
12/2/24	CA	Stanchion	ND
5/13/24	CO	office desks	ND
5/13/24	CO	Office door handles	ND
5/13/24	CO	Office sink and refrigerator	ND
5/13/24	CO	Break room	ND
5/13/24	CO	Restroom	ND
5/13/24	CO	Medicine room	ND
5/13/24	CO	Drop hoses	ND
5/13/24	CO	Inflation/teat liner	ND
5/13/24	CO	Milking units	ND
5/13/24	CO	Stanchion	ND
5/13/24	CO	Stanchion floor	ND
5/13/24	CO	Milk lines	ND
5/13/24	CO	Gloves	ND
5/13/24	CO	Washing machine	ND
5/13/24	CO	Stair rails	ND
5/13/24	CO	Boot wash area	ND
5/13/24	CO	Milk filters	34.04
5/13/24	CO	Tank room sink/hose	38.12
5/13/24	CO	Bulk tank hose	33.1
5/13/24	CO	Control panel	ND
5/13/24	CO	Head locks and feed bunk	ND
5/13/24	CO	Waterer	ND
5/13/24	CO	Water tank	ND
5/13/24	CO	Rails in free stalls	ND
5/13/24	CO	Alleway gates	ND
5/13/24	CO	Push up tractor	ND
5/13/24	CO	Sick parlor inflation	34.27
5/13/24	CO	Sick parlor milk line	ND
5/13/24	CO	Sick parlor stanchion floor	ND
5/13/24	CO	sick parlor milk room	ND
5/13/24	CO	Head locks and feed bunk	ND
5/13/24	CO	Feed bunk	ND
5/13/24	CO	Head locks and feed bunk	ND
5/13/24	CO	feed bunk	ND
5/13/24	CO	Waterer	ND
5/13/24	CO	Bedding	ND
5/13/24	CO	Shade supports	ND
5/13/24	CO	Waterer	ND
5/13/24	CO	Bedding	ND
5/13/24	CO	Shade supports	ND
5/8/24	CO	Lockups and feedbunk	ND
5/8/24	CO	Fresh water holding tank	ND
5/8/24	CO	Blue plastic feeder	ND
5/8/24	CO	Feed bunk/head locks	ND
5/8/24	CO	Mineral tub	ND
5/8/24	CO	Head locks/feed bunk	ND
5/8/24	CO	Pitch fork	ND
5/8/24	CO	W fence	ND
5/8/24	CO	Exudate in bedding	ND

5/8/24	CO	Bedding	ND
5/8/24	CO	E fence	ND
5/8/24	CO	Heifer pen	ND
5/8/24	CO	Waterer	ND
5/13/24	CO	Inflation/teat liner	ND
5/13/24	CO	Water and milk lines	ND
5/13/24	CO	Breezway disinfection station	ND
5/13/24	CO	Office door handles and light switches	ND
5/13/24	CO	Gloves	ND
5/13/24	CO	Gloves	ND
5/13/24	CO	Drain	ND
5/13/24	CO	Stanchion floor	ND
5/13/24	CO	Office desk and rails	ND
5/8/24	CO	Lockups and feedbunk	ND
5/13/24	CO	Butt plates	28.17
5/13/24	CO	Parlor Floor	38.17
5/13/24	CO	Parlor water and milk liners	ND
5/13/24	CO	Butt plates	ND
5/13/24	CO	Parlor drain	ND
5/13/24	CO	inflation/teat liner	ND
5/13/24	CO	Breezeway door handles and light switches	ND
5/13/24	CO	inflation/teat liner	ND
5/13/24	CO	Parlor floor	ND
5/13/24	CO	Breezeway doors and hoses	ND
5/8/24	CO	Water tank	ND
5/13/24	CO	Vehicle side by side	ND
5/8/24	CO	Fence to holding pen	ND
5/8/24	CO	Feed push up trailer blade	ND
5/8/24	CO	Feed truck	ND
5/8/24	CO	Lockups and feedbunk	ND
6/26/24	CO	inflation/teat liner	ND
6/26/24	CO	inflation/teat liner	ND
6/26/24	CO	inflation/teat liner	37.36
6/26/24	CO	inflation/teat liner	32.72
6/26/24	CO	inflation/teat liner	ND
6/26/24	CO	inflation/teat liner	ND
6/26/24	CO	Drop hose	ND
6/26/24	CO	Drop hose	ND
6/26/24	CO	Drop hose	ND
6/26/24	CO	Drop hose	ND
6/26/24	CO	Drop hose	ND
6/26/24	CO	Drop hose	ND
6/26/24	CO	Parlor surface	ND
6/26/24	CO	Parlor surface	ND
6/26/24	CO	Parlor surface	ND
6/26/24	CO	Parlor floor	38.87
6/26/24	CO	Parlor floor	ND
6/26/24	CO	parlor drain	39.7
7/2/24	CO	Waterer	ND
7/2/24	CO	Waterer	ND
7/2/24	CO	Waterer	ND
7/2/24	CO	Feed robot	ND
7/2/24	CO	inflation/teat liner	ND

7/2/24	CO	inflation/teat liner	ND
7/2/24	CO	inflation/teat liner	ND
7/2/24	CO	inflation/teat liner	ND
7/2/24	CO	inflation/teat liner	ND
7/2/24	CO	inflation/teat liner	ND
7/2/24	CO	Dip cup	ND
7/2/24	CO	Dip cup	ND
7/2/24	CO	Gloves	ND
7/2/24	CO	Drop hose	ND
7/2/24	CO	Drop hose	ND
7/2/24	CO	Drop hose	ND
7/2/24	CO	Drop hose	ND
7/2/24	CO	Drop hose	ND
7/2/24	CO	Drop hose	ND
7/2/24	CO	Parlor Surface	ND
7/2/24	CO	Parlor Surface	ND
7/2/24	CO	Parlor Surface	ND
7/2/24	CO	Parlor Surface	ND
7/2/24	CO	Parlor Surface	ND
7/2/24	CO	Parlor Surface	ND
7/2/24	CO	Parlor floor	ND
7/2/24	CO	Parlor floor	ND
7/2/24	CO	Parlor floor	ND
7/2/24	CO	Parlor floor	ND
7/2/24	CO	Parlor floor	ND
7/2/24	CO	Parlor floor	ND
7/2/24	CO	Parlor drain	ND
7/9/24	CO	Parlor Surfaces	ND
7/9/24	CO	Water drop hoses	ND
7/9/24	CO	Parlor floor	ND
7/9/24	CO	Milk Hoses	ND
7/9/24	CO	Gloves	ND
7/9/24	CO	Parlor Drain	ND
7/9/24	CO	Machine Shut offs	ND
7/9/24	CO	Gloves	ND
7/9/24	CO	inflation/teat liner	ND
7/9/24	CO	inflation/teat liner	ND
7/9/24	CO	inflation/teat liner	ND
7/9/24	CO	Bedding	ND
7/9/24	CO	Sick pen headlocks	ND
7/9/24	CO	Lagoon East	ND
7/9/24	CO	Lagoon West	ND
7/9/24	CO	Rim of water trough	ND
7/9/24	CO	waterer	ND
7/9/24	CO	waterer	ND
7/9/24	CO	waterer	ND
7/9/24	CO	waterer	ND
8/6/24	CO	waterer	ND
8/20/24	CO	inflation/teat liner	ND
8/20/24	CO	inflation/teat liner	ND
8/20/24	CO	inflation/teat liner	ND
8/20/24	CO	inflation/teat liner	ND
8/22/24	CO	inflation/teat liner	ND

8/22/24	CO	inflation/teat liner	ND
8/22/24	CO	inflation/teat liner	ND
8/22/24	CO	inflation/teat liner	ND
8/22/24	CO	inflation/teat liner	ND
8/22/24	CO	inflation/teat liner	ND
8/22/24	CO	inflation/teat liner	ND
8/22/24	CO	inflation/teat liner	ND
8/22/24	CO	inflation/teat liner	ND
8/22/24	CO	inflation/teat liner	ND
8/22/24	CO	inflation/teat liner	ND
8/22/24	CO	inflation/teat liner	ND
8/22/24	CO	inflation/teat liner	ND
8/22/24	CO	inflation/teat liner	ND
8/22/24	CO	inflation/teat liner	ND
8/22/24	CO	inflation/teat liner	ND
8/22/24	CO	inflation/teat liner	ND
8/22/24	CO	inflation/teat liner	ND
7/31/24	CO	inflation/teat liner	ND
7/31/24	CO	inflation/teat liner	ND
7/31/24	CO	inflation/teat liner	ND
7/31/24	CO	inflation/teat liner	ND
7/31/24	CO	Parlor drain	ND
7/31/24	CO	Inflation/teat liner	38.74
7/16/24	CO	Inflation/teat liner	39.18
7/16/24	CO	Inflation/teat liner	30.69
7/16/24	CO	Inflation/teat liner	ND
7/16/24	CO	Inflation/teat liner	28.07
7/16/24	CO	Inflation/teat liner	ND
7/16/24	CO	Inflation/teat liner	32.56
7/16/24	CO	Inflation/teat liner	ND
7/16/24	CO	Inflation/teat liner	32.86
7/16/24	CO	Inflation/teat liner	31.49
7/16/24	CO	Inflation/teat liner	33.43
7/16/24	CO	Inflation/teat liner	31.9
7/16/24	CO	Inflation/teat liner	ND
7/16/24	CO	Inflation/teat liner	38.4572
7/16/24	CO	Inflation/teat liner	ND
7/16/24	CO	Inflation/teat liner	29.19
7/16/24	CO	Inflation/teat liner	35.24
7/16/24	CO	Inflation/teat liner	ND
7/16/24	CO	Inflation/teat liner	32.24
7/16/24	CO	Inflation/teat liner	ND
12/16/24	CA	Inflation/teat liner	36.9
12/16/24	CA	Inflation/teat liner	34.7
12/16/24	CA	Inflation/teat liner	28.9
12/16/24	CA	Inflation/teat liner	30.59
12/18/24	CA	mineral block	ND
12/18/24	CA	mineral block	ND
12/19/24	CA	Inflation/teat liner	34.1
12/19/24	CA	Inflation/teat liner	30.8
12/19/24	CA	Inflation/teat liner	ND
12/19/24	CA	Inflation/teat liner	26.5
12/19/24	CA	milking unit	38.7

12/19/24	CA	milking unit	ND

[1]ND stands for non-detected
